# Improving MR image quality with a multi-task model, using convolutional losses

**DOI:** 10.1186/s12880-023-01109-z

**Published:** 2023-10-02

**Authors:** Attila Simkó, Simone Ruiter, Tommy Löfstedt, Anders Garpebring, Tufve Nyholm, Mikael Bylund, Joakim Jonsson

**Affiliations:** 1 Department of Radiation Sciences, Umeå University, Umeå, Sweden; 2 Department of Biomedical Engineering, Eindhoven University of Technology, Eindhoven, The Netherlands; 3 Department of Computing Science, Umeå University, Umeå, Sweden

**Keywords:** Machine learning, Magnetic resonance imaging, Image artefact correction

## Abstract

**Purpose:**

During the acquisition of MRI data, patient-, sequence-, or hardware-related factors can introduce artefacts that degrade image quality. Four of the most significant tasks for improving MRI image quality have been bias field correction, super-resolution, motion-, and noise correction. Machine learning has achieved outstanding results in improving MR image quality for these tasks individually, yet multi-task methods are rarely explored.

**Methods:**

In this study, we developed a model to simultaneously correct for all four aforementioned artefacts using multi-task learning. Two different datasets were collected, one consisting of brain scans while the other pelvic scans, which were used to train separate models, implementing their corresponding artefact augmentations. Additionally, we explored a novel loss function that does not only aim to reconstruct the individual pixel values, but also the image gradients, to produce sharper, more realistic results. The difference between the evaluated methods was tested for significance using a Friedman test of equivalence followed by a Nemenyi post-hoc test.

**Results:**

Our proposed model generally outperformed other commonly-used correction methods for individual artefacts, consistently achieving equal or superior results in at least one of the evaluation metrics. For images with multiple simultaneous artefacts, we show that the performance of using a combination of models, trained to correct individual artefacts depends heavily on the order that they were applied. This is not an issue for our proposed multi-task model. The model trained using our novel convolutional loss function always outperformed the model trained with a mean squared error loss, when evaluated using Visual Information Fidelity, a quality metric connected to perceptual quality.

**Conclusion:**

We trained two models for multi-task MRI artefact correction of brain, and pelvic scans. We used a novel loss function that significantly improves the image quality of the outputs over using mean squared error. The approach performs well on real world data, and it provides insight into which artefacts it detects and corrects for. Our proposed model and source code were made publicly available.

## Background

Due to the sensitivity of the magnetic resonance imaging (MRI) data acquisition process, slight changes around the scanner system can produce artefacts in the images. The artefacts generally belong to three main classes: hardware-related (e.g. magnetic field inhomogeneities, zipper artefacts, bias fields), sequence-related (e.g. aliasing, subsampled *k*-space, Gibbs-ringing, low signal-to-noise ratio (SNR) due to short acquisition time) or patient-related (e.g. involuntary patient motion) [1, 2].

Four of the most significant, often addressed MRI artefacts have been identified as: bias fields, subsampled *k*-space, patient motion, and noise. Proposed machine learning solutions commonly establish state-of-the-art results [3–6] for correcting these artefacts individually. However, when training a model for an individual task, it most commonly does not include any other artefacts. For example bias field correction is often accounted for as a pre-processing step in models for other tasks [7, 8] instead of something to consider by the model. In real-life scenarios, multiple artefacts can appear simultaneously, hindering the performance of such models. Multi-task learning offers a solution and it has been shown to result in more robust models [9], yet it has only been applied to MRI artefact correction in a few cases [10, 11], where multiple artefacts are involved in the training dataset, however the model architecture does not separate the corrections according to what artefact they correspond to.

Common conclusions from research in correcting these individual artefacts have shown that after training on artificially augmented artefacts, the performance translates well to real-life data [12–14]. Furthermore, meaningful corrections can be performed in both the image [15] and the Fourier space (*k*-space) [16] of the data. A recently proposed framework for MRI super-resolution [17] exploits the fact that the problem can be viewed both in the image and Fourier space by implementing Fourier transform layers in the model architecture, which has recently been implemented to reconstruct MRI images [11].

In machine learning research for medical imaging, the most common choices for loss functions include minimising the pixel-wise $L_1$ or $L_2$ losses, since they are easy to compute and usually lead to quick convergence [18]. However, it is becoming increasingly clear that they have a low correlation to perceptual quality [19, 20]. Alternative loss functions have recently been proposed for more robust results [21] or to show better correlation to the perceptual quality of the images. This can be achieved by using the deep features of the model in the loss [22], or by using an adversarial model in a GAN setting [23], but these tend to increase the complexity of training the model, and the self-supervision of GANs introduces other concerns such as hallucinations [24]. Alternatively, making small modifications such as including the image gradients in the loss function has shown to benefit the sharpness of the predictions [25, 26].

In the presented work, we investigate a multi-task model, trained using a novel loss function, and how it performs the task of MRI artefact correction, compared to a combination of approaches that handle only a single task. Our main contributions can be divided into the following components:

- Using a multi-task learning approach, our model is trained to correct for four types of artefacts simultaneously, even though it has not encountered an image with multiple artefacts during training.
- Our model was trained using a novel loss function based on convolutional kernels that is simple to compute, yet it contains image gradient information that could help the model reconstruct images of better perceptual quality.
- Our implementations of the artefact augmentations and the trained models are publicly available.

## Methodology

We begin by discussing our model architecture, followed by a summary of the datasets used. Then we detail our augmentations of four artefacts in MRI imaging, identified as some of the most significant: bias fields, subsampled *k*-space, patient motion, and noise. This is followed by the proposed alternative loss function.

### Model

Model architectures that incorporate the *k*-space information as well as image space information about the input image have been shown to perform well for MR image reconstruction tasks [16, 17], therefore we have decided to implement an interleaved model architecture that simultaneously corrects the images in both domains [11].

Our baseline architecture is the SRResNet [27]—originally introduced for super-resolution tasks,—due to its photo-realistic reconstructions. To exploit both the image and frequency domains of the image, we used the combination of two SRResNet models. The first model operates on the input image, and outputs the residuals in the image space. Alternatively, the second model implements a Fourier transform layer on the input image, which means the model operates on the *k*-space of the data. Additionally, a final layer performing an inverse Fourier transform was also added to the outputs of this model. The outputs of the two models, both in the image space, were added together, which form the outputs of our proposed architecture.

Both models contain twelve convolutional blocks, starting with five strided blocks for downsampling, and ending with five blocks with upsampling layers. The output of the strided blocks is also concatenated with the input of the upsampling blocks, following the U-Net architecture [28].

Each block at downsampling depth *d* contains three 2D convolutional layers with $64 \cdot 2^d$ channels, where $d=\{0, \ldots , 4\}$, with a kernel size of three. These are followed by batch normalization layers and a LeakyReLU activation with a slope of 0.2.

Four individual convolutional layers with a filter size of 1 provide the model outputs. The outputs of the two models are added together providing the four final output residuals of our proposed model. Each output corresponds to a residual, $p_i$, for $i\in \{1,2,3,4\}$, for an artefact. The reconstructed image, *v*, is obtained by adding all residuals to the input image *u*, as $v = u + \sum _i p_i$. A Z-normalization layer is applied on the reconstructed image.

An $\mathcal {L}_2$ regularization term was also added to each of the final output residuals *p*, defined as

1 $$\begin{aligned} \mathcal {L}_2(x) = \alpha \cdot \Vert x \Vert _2^2. \end{aligned}$$

As the regularization term penalizes the residuals of each artefact, it encourages the model to keep the residual small, i.e. keep the input image unchanged. The parameter $\alpha$ determines the balance between the regularization term and the main objective loss. Our implementation of the model has input and output sizes of $320\times 320$. The model returns four residuals, for the four artefacts: bias fields, *k*-space subsampling, motion, and noise. The model has a total of 75.4 million parameters.

### Data

We have performed two sets of experiments: first, the models were evaluated quantitatively performing artefact correction of brain scans using a public dataset for training, validation, and testing. Second, a model was trained and validated on an in-house pelvic MRI dataset, which was evaluated qualitatively on a publicly available dataset for reproducibility. The datasets are further described below.

#### Datasets

**Brain** The public dataset contains 3 969 scans of 3T brain MR images [29, 30] of size $320\times 320$, introduced for the fastMRI challenge [29, 30]^1^ for the task of MRI super-resolution. The dataset was split into training, validation, and testing datasets, using 70, 10, and $20\%$ of the patients, respectively.

**Pelvic** An in-house dataset that includes $T_2$-weighted pelvic MR images from 375 patients captured using a GE Signa 3T PET/MR (GE Healthcare, Chicago, Illinois, United States) at the University Hospital of Umeå, Sweden. The images were $512\times 512$ with 131 slices per patient. Each image slice was cropped in the Fourier space, to yield a $320\times 320$ image. The dataset was split into training, and validation datasets, using 80 and $20\%$ of the patients respectively.

For testing, we used the $T_2$ weighted images from the publicly available Gold Atlas dataset [31]^2^. Similarly, the image slices were $512\times 512$, therefore each slice was cropped in the Fourier space, to size $320\times 320$.

### Artefacts

The training and validation datasets of both the brain and pelvic scans were augmented to obtain images with a varied set of MRI corruptions. Four different types of artefacts were implemented: bias fields, *k*-space subsampling, motion, and noise. To each input image, only one of the augmented artefacts with randomized parameters were applied. We now describe these augmentations in turn.

#### Bias field removal

The term bias field can refer to the effect of various artefacts, for example caused by a non-uniformity in the $\textrm{B}_0$ static field and the transmitted $\textrm{B}_1$ field [1], the inhomogeneity of the RF receive coil [32] or heterogeneous $\textrm{B}_1$ fields. Extensive research on the characteristics of bias fields [33] shows that despite its complex combination of origins, a bias field can be described as a low-frequency multiplicative imaging artefact causing a smooth intensity variation spatially across the image [34–36]. The bias fields were generated in the same way as done by Simkó et al. [36], i.e. we simulated each bias field as a spatial random field (SRF) [37]. For each field, a Gaussian covariance model was used, defined by

2 $$\begin{aligned} \textrm{cov}(r) = \exp \left( -\frac{\pi }{4} \cdot \Big (\frac{r}{l}\Big )^{2}\right) , \end{aligned}$$

where *r* is the distance from a randomly chosen peak of the Gaussian and *l* a length scale relating to the frequency of the field. In the covariance model we used a variance of 50 and a length scale of $10< l < 50$, after downscaling the image to a size of $32 \times 32$ for computational efficiency.

Bias fields can cause an intensity variation in the range of 10% to 40% [34, 35]. In the paper proposing N4ITK, a commonly used correction method [38], bias field ranges of 20% and 40% were used for evaluation. However, we used larger bias field ranges, chosen randomly between 10% and 100%, corresponding to absolute minimum and maximum values between [0.95, 1.05] and [0.5, 2], respectively. This was done to ensure that the bias artefacts introduced a similar degradation in MSE as the other artefact types.

#### *k*-space super-resolution

The fully sampled *k*-space data was subsampled retrospectively by selecting only part of the *k*-space lines (frequencies) using subsampling masks. We used the sampling masks proposed in the fastMRI challenge [30] and also added our own centered masks selecting only the center of the *k*-space. All masks are based on Cartesian sampling, which means they follow a rectilinear pattern.

For the fastMRI masks, *k*-space subsampling was only performed in the phase-encoding direction. When acquiring *k*-space data, frequency-encoding (FE) and phase-encoding (PE) gradients are applied in perpendicular directions to specify the location of the signal. Consecutive steps in the FE direction can be measured with a single radiofrequency pulse, whereas for the PE direction a different radiofrequency pulse needs to be applied for each step. Consequently, PE takes more time than FE and is therefore more susceptible to movement artefacts. For the axial brain scans from the fastMRI dataset the PE direction was from left to right (LR). In this study, the PE direction was chosen randomly for each image on both subsampling types.

The purpose of *k*-space subsampling is to accelerate image acquisitions. For example, when selecting only half of the *k*-space lines, the acquisition time is halved, meaning an acceleration factor of 2. Here the total acceleration factor was chosen uniformly at random to be 2, 3, 4, or 8 to combine the acceleration factors used in the fastMRI dataset [30]. Depending on this acceleration factor, a band of 16, 12, 8, or 4 % of the total *k*-space lines was included in the centre of the fastMRI masks to maintain the low-frequency information of the image. The remaining *k*-space lines were uniformly chosen to be sampled either equidistantly or randomly. The equidistant sampling began with a random offset from the start, so that first a few *k*-space lines could be skipped before the sampling began. For the centered masks, only the centre square of *k*-space data was kept. The width of this square was again 16, 12, 8, or 4 % of the total *k*-space width depending on the selected acceleration factor.

#### Motion correction

We added rigid motion to the brain scans, and both rigid and periodic motion to the pelvic scans.

Rigid motion corrupted scans were simulated by adding rigid transformations to image space over a series of timesteps. First, the number of *k*-space lines to be corrupted was uniformly selected between 30 to 80% of the total *k*-space lines, and these lines were then split randomly into 4–24 segments. A piece-wise constant approach was used, where all lines within a segment were corrupted with the same motion parameters. These rotation and translation parameters were sampled from a Gaussian distribution with zero mean and standard deviations of 12$^{\circ }$ and 30 voxels respectively. These parameters were then applied to the image space and the corrupted image was converted back to *k*-space. The *k*-space lines belonging to that segment were sampled and these segments were then combined to form the motion corrupted *k*-space.

The periodic motion, which was added to simulate breathing, was implemented similar to Tamada et al. [39]. A phase error was added to the *k*-space in the PE direction, for the pelvic images being AP. The corrupted *k*-space signal *S* was given by

3 $$\begin{aligned} S(k_{x}, k_{y}) = S_{0}(k_{x}, k_{y})e^{-j\phi (k_{y})}, \end{aligned}$$

where $S_{0}$ is the original *k*-space signal, $k_{x}$ and $k_{y}$ the FE and PE directions with $-\pi \le k_{x} \le \pi$ and $-\pi < k_{y} \le \pi$, and $\phi (k_{y})$ the phase error. The phase error is defined as,

4 $$\begin{aligned} \phi (k_{y}) = \left\{ \begin{array}{ll} k_{y}\Delta \sin (\alpha k_{y} + \beta ), &{} \text {if}\ \left| k_{y} \right| > k_{y_{0}}, \\ 0, &{} \text {otherwise,} \\ \end{array}\right. \end{aligned}$$

where $k_{y_{0}}$ is the range of centre *k*-space lines to which no motion was added with $\frac{\pi }{10} < k_{y_{0}} \le \frac{\pi }{8}$. This was to keep the corrupted image better aligned with the clean image. The $\Delta$ is the extent of the motion in pixels with $20 \le \Delta \le 120$, the $\alpha$ is the period of the respiratory wave in Hz with $0.1 \le \alpha \le 5$, and $\beta$ is the phase of the respiratory wave with $0 \le \beta \le \frac{\pi }{4}$. For each of these four parameters the exact values were uniformly selected between the specified ranges. We used smaller values for $k_{y_{0}}$ and larger values for $\Delta$ than Tamada et al. [39], to simulate larger motion artefacts.

#### Noise removal

We corrupted the *k*-space of the relatively noise-free images with complex Gaussian white noise such that the SNR of the *k*-space decreased to a certain value. The following definition of SNR was used similar to Cohen et al. [40],

5 $$\begin{aligned} \textrm{SNR} = 20log _{10}\left( \frac{S}{N}\right) , \end{aligned}$$

with *S* being the mean absolute *k*-space value and *N* the standard deviation of the added noise, which was equal for the real and imaginary components. The designated SNR for each *k*-space was selected uniformly in the range $[-12, 10]$ dB based on visual inspection of the noise levels.

### Loss functions

#### Multi-task

We implemented a multi-task loss function [41] to correct multiple artefacts simultaneously. For each sample in the training data, the loss function would take into account which artefact was used to corrupt it, and the model only minimized the loss with respect to the output residuals to the corresponding artefact, and ignored the other residuals. The loss was,

6 $$\begin{aligned} \mathcal {L}_{\textrm{total}}(v, u, p, y) = \mathcal {L}(v, u + \sum _{i=1}^n \lambda [y = i]p_i), \end{aligned}$$

where *v* denotes the artefact-free image, *u* denotes the image with added artefacts, *p* is the list of residuals for each of the $n = 4$ artefacts, and *y* is the index of the artefact that was used in the current training sample. Each residual was added to the output depending on the indicator function $\lambda [y = i]$, which returns 1 if *y* equals *i*, and 0 otherwise. The indicator function sets the unknown residuals to zero in the loss function.

#### Perceptual loss

In the area of image restoration with machine learning solutions, new models are often introduced that outperform the state-of-the-art methods, but the loss functions used when training such models have received less attention.

The *MSE* loss is often used in regression problems due to its simplicity, well-understood statistical interpretation, fast convergence, and low computational cost [18]. However, since the *MSE* assumes independent Gaussian distributed errors, it often results in a loss of contrast. The *MSE* loss has also been shown to have a low correlation with perceptual quality [19].

The *MSE* loss regards the difference between pixel intensities. What we propose is to also include information about the relationship between neighbouring pixels, in the form of image gradients, without significantly increasing the complexity of the loss computation. We begin by looking at the *MSE* loss,

$$\begin{aligned} MSE(x, \hat{x}) = \frac{1}{N} \sum _{j=0}^{N} (x - \hat{x})_j^2, \end{aligned}$$

where $x - \hat{x}$ contains the residual matrix between the true and predicted images, *j* is the image pixel index, and *N* is the number of image pixels.

To this definition, we propose to introduce a convolutional kernel *I*, that is applied on both images before taking their element-wise difference, introducing the proposed convolutional loss $\mathcal {L}$ as,

$$\begin{aligned} \mathcal {L}(I, x, \hat{x})&= \frac{1}{N} \sum _{j=0}^{N} (I*x - I*\hat{x})_j^2\\&= MSE(I * x, I * \hat{x}), \end{aligned}$$

where $*$ is a (discrete) convolution. If *I* equals the identity kernel (e.g. $3\times 3$ $I_E$, seen in Table 1) the proposed convolutional loss function is identical to *MSE*. Looking at the kernel $I_E$, it comes as no surprise, that only the differences between the pixel values are considered, and their relationship to their neighbouring pixels are disregarded.

**Table 1:** The list of kernels that are used in $\mathcal {L}_{C}$, our proposed convolutional loss

Name	Kernel
$I_{E}$	$$\left[ {\begin{array}{rrr} 0 &{} 0 &{} 0 \\ 0 &{} 1 &{} 0 \\ 0 &{} 0 &{} 0 \\ \end{array}} \right]$$
$I_{PT}$	$$\left[ {\begin{array}{rrr} -1 &{} -1 &{} -1 \\ 0 &{} 0 &{} 0 \\ 1 &{} 1 &{} 1 \\ \end{array}} \right]$$
$I_{PR}$	$$\left[ {\begin{array}{rrr} 1 &{} 0 &{} -1 \\ 1 &{} 0 &{} -1 \\ 1 &{} 0 &{} -1 \\ \end{array}} \right]$$
$I_{S3T}$	$$\left[ {\begin{array}{rrr} -1 &{} -2 &{} -1 \\ 0 &{} 0 &{} 0 \\ 1 &{} 2 &{} 1 \\ \end{array} } \right]$$
$I_{S3R}$	$$\left[ {\begin{array}{rrr} 1 &{} 0 &{} -1 \\ 2 &{} 0 &{} -2 \\ 1 &{} 0 &{} -1 \\ \end{array}}\right]$$
$I_{S5T}$	$$\left[ {\begin{array}{rrrrr} -2 &{} -2 &{} -4 &{} -2 &{} -2 \\ -1 &{} -1 &{} -2 &{} -1 &{} -1 \\ 0 &{} 0 &{} 0 &{} 0 &{} 0 \\ 1 &{} 1 &{} 2 &{} 1 &{} 1 \\ 2 &{} 2 &{} 4 &{} 2 &{} 2 \\ \end{array} } \right]$$
$I_{S5R}$	$$\left[ {\begin{array}{rrrrr} 2 &{} 1 &{} 0 &{} -1 &{} -2 \\ 2 &{} 1 &{} 0 &{} -1 &{} -2 \\ 4 &{} 2 &{} 0 &{} -2 &{} -4 \\ 2 &{} 1 &{} 0 &{} -1 &{} -2 \\ 2 &{} 1 &{} 0 &{} -1 &{} -2 \\ \end{array} } \right]$$
$I_{L3}$	$$\left[ {\begin{array}{rrr} 0 &{} -1 &{} 0 \\ -1 &{} 4 &{} -1 \\ 0 &{} -1 &{} 0 \\ \end{array} } \right]$$
$I_{L5}$	$$\left[ {\begin{array}{rrrrr} 0 &{} 0 &{} -1 &{} 0 &{} 0 \\ 0 &{} -1 &{} -2 &{} -1 &{} 0 \\ -1 &{} -2 &{} 16 &{} -2 &{} -1 \\ 0 &{} -1 &{} -2 &{} -1 &{} 0 \\ 0 &{} 0 &{} -1 &{} 0 &{} 0 \\ \end{array} } \right]$$

We propose an extension of this loss function, by adding various kernels to replace $I_E$. As an example, using edge detection kernels as *I* means that the loss will focus on, by design, high contrast changes between the pixels. Our proposed convolutional loss function uses a combination of nine kernels, defined as:

$$\begin{aligned} \mathcal {L}_{C}(x, \hat{x})&= \delta _E \mathcal {L}(I_{E}, x, \hat{x}) \\&\quad + \delta _{PT} \mathcal {L}(I_{PT}, x, \hat{x}) + \delta _{PR} \mathcal {L}(I_{PR}, x, \hat{x}) \\&\quad + \delta _{S3T} \mathcal {L}(I_{S3T}, x, \hat{x}) + \delta _{S3R} \mathcal {L}(I_{S3R}, x, \hat{x}) \\&\quad + \delta _{S5T} \mathcal {L}(I_{S5T}, x, \hat{x}) + \delta _{S5R} \mathcal {L}(I_{S5R}, x, \hat{x}) \\&\quad + \delta _{L3} \mathcal {L}(I_{L3}, x, \hat{x}) + \delta _{L5} \mathcal {L}(I_{L5}, x, \hat{x}) \end{aligned}$$

where each kernel is collected in Table 1, and each component of the loss has a scaling factor $\delta$. We have collected the Prewitt top ($I_{PT}$) and right ($I_{PR}$) operators and the Sobel operators for both the $3\times 3$ and $5\times 5$ case ($I_{S3T}$, $I_{S3R}$ and $I_{S5T}$, $I_{S5R}$ respectively) which are often used for edge detection, and also both the $3\times 3$ and $5\times 5$ Laplace operators ($L_3$ and $L_5$, respectively). We tune their corresponding $\delta$ values during optimization to explore which components the models find most beneficial.

$\mathcal {L}_{C}$ implements convolutions to incorporate the image gradients in the computed loss, while the optimization of the scaling factors allows the model to show which kernels make a significant contribution to the model performance. Once the optimal scaling factors are found, the proposed loss does not significantly increase the computational complexity of the loss over *MSE*, only introducing convolutional operations which can be performed on a GPU.

## Experiments

To evaluate the effect of our multi-task approach and the convolutional losses, we have trained seven different models.

As a benchmark on how well the proposed model architecture performs for correcting the individual tasks, we have trained four models using the brain scans for correcting only one of the artefacts: bias, subsampling, motion, and noise. For these models, the architecture was modified to return only one residual. The corresponding models are denoted *Bias*, *Subsampling*, *Motion*, and *Noise*, respectively.

A model was also trained on the brain scans using the multi-task approach, correcting for all artefacts simultaneously, denoted *MT*.

Additionally, we have also trained a model using the multi-task approach, as well as the proposed convolutional loss, denoted *MT+*$\mathcal {L}_C$.

Finally, a multi-task model using the convolutional loss was also trained on the pelvic dataset, denoted *Pelvis MT+*$\mathcal {L}_C$.

The models were first evaluated and compared on their performance of correcting the individual artefacts. Afterwards the models were evaluated on how well they perform if multiple artefacts are present simultaneously. The *Pelvis MT+*$\mathcal {L}_C$ model was then evaluated qualitatively on the Gold Atlas dataset.

We selected the evaluation metrics based on the findings of Mason et al. [19] looking into the gap between commonly used image quality metrics and expert human evaluations of image quality. Despite their low correlation to perceptual quality, we used the metrics of *MSE* and *SSIM* due to their popularity, and we have also used the more complex Visual Information Fidelity (*VIF*) metric [42] since it has been shown to correlate well with human perceptual quality. The difference between the evaluated methods was tested for significance using a Friedman test of equivalence followed by a Nemenyi post-hoc test [43] with a threshold of 0.05.

### Bayesian hyper-parameter search

A Bayesian optimization was performed to find the best set of model hyper-parameters. This method was selected over random or grid search methods due to its better efficiency as it typically requires fewer iterations to find the optimal hyper-parameters [44]. The training of each model was stopped when the performance on the validation dataset did not improve for 10 epochs, using *MSE* as the validation metric. The Bayesian process had 35 iterations for the models implementing the convolutional loss (*MT+*$\mathcal {L}_C$ and *Pelvis MT+*$\mathcal {L}_C$), and 10 iterations for all other models, as the convolutional loss introduces 9 additional parameters to optimize.

We performed the Bayesian optimization over optimization algorithms (Adam [45] and RMSprop [46]), learning rates ($10^\gamma$, selecting $\gamma$ from the range [-7, -2]), regularization parameters (from the range [0, 1]), and scaling factors (from the range [0, 1]), with the optimized values collected in Table 2.

**Table 2:** Results of the Bayesian hyper-parameter optimization. The values marked with “$^*$” were not optimized, since the $\delta$ parameters are only used in the models that implement the convolutional loss ($\mathcal {L}_C$)

Hyper-p.	Models						
	*Bias*	*Subsampling*	*Motion*	*Noise*	*MT*	*MT+*$\mathcal {L}_C$	*Pelvis MT+*$\mathcal {L}_C$
Optim.	RMSprop	RMSprop	RMSprop	RMSprop	RMSprop	RMSprop	RMSprop
L.r., $10^{\gamma }$	$-3.79$	$-4.39$	$-4.17$	$-4.06$	$-3.73$	$-3.91$	$-3.88$
$\alpha$	0.60	0.48	0.59	0.85	0.51	0.87	0.89
$\delta _{E}$	$1^*$	$1^*$	$1^*$	$1^*$	$1^*$	0.30	0.13
$\delta _{PT}$	$0^*$	$0^*$	$0^*$	$0^*$	$0^*$	0.83	0.23
$\delta _{PR}$	$0^*$	$0^*$	$0^*$	$0^*$	$0^*$	0.25	0.03
$\delta _{S3T}$	$0^*$	$0^*$	$0^*$	$0^*$	$0^*$	0.74	0.84
$\delta _{S3R}$	$0^*$	$0^*$	$0^*$	$0^*$	$0^*$	0.30	0.23
$\delta _{S5T}$	$0^*$	$0^*$	$0^*$	$0^*$	$0^*$	0.21	0.14
$\delta _{S5R}$	$0^*$	$0^*$	$0^*$	$0^*$	$0^*$	0.72	0.91
$\delta _{L3}$	$0^*$	$0^*$	$0^*$	$0^*$	$0^*$	0.09	0.38
$\delta _{L5}$	$0^*$	$0^*$	$0^*$	$0^*$	$0^*$	0.82	0.77

### Evaluating the artefact corrections

The performance of each individual task was investigated, with the trained models being compared to analytical or established machine learning solutions proposed for the individual tasks. Keep in mind, that while most of the methods for comparison can only be applied to a single task, the *MT* and *MT+*$\mathcal {L}_C$ models are the same for all experiments.

#### Bias field correction

The models trained for bias field correction (*Bias*, *MT*, and *MT+*$\mathcal {L}_C$) were compared to the N4ITK method provided by the SimpleITK package [38] and to a machine learning-based bias field correction model, described by Simkó et al. [36].

We applied artificial bias fields of $5\%$, $10\%$, and $20\%$—which correspond to normalizing the bias fields between [0.95, 1.15], [0.9, 1.1], and [0.8, 1.2], respectively—and compared the corrections of the three methods using *SSIM* and *VIF*.

The N4ITK method was optimized for use on the testing dataset without downsampling and using 4 control points. The input image intensities were scaled to a range between 0 and 10.

#### Subsampling the *k*-space

We compared our three models trained for improving the resolution of images (*Subsampling*, *MT*, and *MT+*$\mathcal {L}_C$) to bicubic upsampling and UniRes^3^ [47, 48] a machine learning solution for improving the resolution of MRI images. Their performance is evaluated on the brain scans from the testing dataset. Here each image slice was downsampled using three acceleration factors ($\times 2$, $\times 3$, and $\times 4$) with the centered masks. Selecting only the center of the *k*-space allows for removing all the values of the *k*-space excluded by the mask leading to a smaller downsampled image size, which allows comparisons to bicubic interpolation that takes an image with a smaller image size and increases it to the original size.

#### Motion

For motion artefact correction, our three corresponding trained models (*Motion*, *MT*, and *MT+*$\mathcal {L}_C$) was compared to Total Variation Denoising (TV), following the work in [49], available from the scikit-image [50] Python library. Performing a Bayesian optimization on the weighting parameter with the range $(0.01, 0.02, \ldots , 0.15)$, the best results were achieved with 0.05. We evaluate the methods for three percentages of the center of the *k*-space that was not altered by the artefact augmentation: $25\%$, $12.5\%$, and $5\%$. This means motion artefacts were not introduced in the center $25\%$ of the image in the first experiment, $12.5\%$ for the second, and $5\%$ for the last.

#### Noise

For noise removal, our three corresponding trained models (*Noise*, *MT*, and *MT+*$\mathcal {L}_C$) were compared to a curvature anisotropic diffusion denoising algorithm from the SimpleITK Python package^4^ with time step 0.0625 and 5 iterations and using BM3D^5^ [51] with hard thresholding and $\sigma = 0.2$. We evaluate the methods for three *SNR* values: 5, 0, and $-5$ dB.

### Evaluating multi-task learning

After evaluating the models for the individual tasks, the multi-task approach is further evaluated on a dataset where multiple artefacts are present simultaneously. All artefact combinations have been evaluated, and they demonstrate comparable results, hence only two particularly interesting combinations are presented. In the first scenario, we applied both subsampling and motion artefacts on the brain scans from the testing dataset. Although the background of the two artefacts are very different, they both create a blurry effect, and cause ringing artefacts, making it more difficult for the model to correct individually. The two artefacts were applied in a random order for each slice. We evaluated how applying the *Subsampling* and *Motion* models consecutively compared to the performance of the multi-task models (*MT* and *MT+*$\mathcal {L}_C$).

In the second scenario, we applied noise and bias fields on the available brain scans of the testing dataset. Since we have added complex Gaussian white noise, the model might always assume that the noise comes from this distribution. However as the bias field adds a multiplicative noise over the image, the distribution of the noise term changes. The two artefacts were applied in a random order for each slice. We evaluated how applying the *Noise* and *Bias* models consecutively compared to the performance of the multi-task models (*MT* and *MT+*$\mathcal {L}_C$).

An inherent quality of the multi-task models is the residual outputs with respect to each artefact correction. First, this allows to disregard any of the corrections if not all artefacts are to be corrected. Second, the mean absolute value of the four outputs gives insight into how much each of the artefacts are corrected for. We collected the mean absolute value of the output residuals of the multi-task models for both scenarios.

### Qualitative evaluation

To explore the performance on real world datasets with simultaneous artefacts, the model trained on pelvic datasets, using the multi-task approach and the convolutional loss function was evaluated on the Gold Atlas dataset. We applied the trained model (*Pelvis MT+*$\mathcal {L}_C$) on scans from two patients.

## Results

The Bayesian hyper-parameter optimization found that all seven models achieved the best results using the RMSprop optimizer, with the optimal learning rates and $\alpha$ values collected in Table 2.

The performance of the trained models for individual artefact correction is compared to other established methods. For bias field correction, the results are collected in Table 3. For *k*-space subsampling, the results can be found in Table 4, while for motion and noise correction, the results are collected in Tables 5 and 6, respectively.

**Table 3:** The results and their standard errors for bias field correction. The three experiments differ in the selected magnitude of the applied bias fields. The performance of the selected models is compared using regards to *SSIM* and *VIF*. The results in bold indicate the best performance without significant differences between them following a Nemenyi post-hoc test

		SSIM	VIF
$5\%$	Original	$0.973\pm 0.018$	$1.024\pm 0.016$
	N4ITK	$0.972\pm 0.033$	$\varvec{1.050\pm 0.093}$
	Implicit	$0.978\pm 0.022$	$1.003\pm 0.032$
	*Bias*	$\varvec{0.986\pm 0.013}$	$1.011\pm 0.032$
	*MT*	$\varvec{0.983\pm 0.020}$	$1.019\pm 0.040$
	*MT+*$\mathcal {L}_C$	$0.978\pm 0.020$	$\varvec{1.043\pm 0.045}$
$10\%$	Original	$0.957\pm 0.043$	$1.022\pm 0.038$
	N4ITK	$0.969\pm 0.034$	$\varvec{1.058\pm 0.098}$
	Implicit	$0.954\pm 0.044$	$1.021\pm 0.045$
	*Bias*	$\varvec{0.982\pm 0.016}$	$1.016\pm 0.037$
	*MT*	$\varvec{0.979\pm 0.021}$	$1.024\pm 0.043$
	*MT+*$\mathcal {L}_C$	$\varvec{0.976\pm 0.022}$	$\varvec{1.044\pm 0.047}$
$20\%$	Original	$0.931\pm 0.066$	$1.012\pm 0.064$
	N4ITK	$0.965\pm 0.035$	$\varvec{1.072\pm 0.103}$
	Implicit	$0.950\pm 0.067$	$1.050\pm 0.066$
	*Bias*	$\varvec{0.981\pm 0.020}$	$1.022\pm 0.044$
	*MT*	$\varvec{0.978\pm 0.025}$	$1.030\pm 0.046$
	*MT+*$\mathcal {L}_C$	$0.976\pm 0.023$	$1.042\pm 0.049$

**Table 4:** The results and their standard errors for super-resolution. The three experiments differ in the acceleration factor of the subsampling of the images. The performance of the selected models is compared using regards to *SSIM* and *VIF*. The results in bold indicate the best performance without significant differences between them following a Nemenyi post-hoc test

		SSIM	VIF
$\times 2$	Bicubic	$0.668\pm 0.146$	$1.001\pm 0.160$
	Zero-filled	$0.830\pm 0.100$	$\varvec{1.007\pm 0.059}$
	UniRes	$0.832\pm 0.191$	$0.863\pm 0.266$
	*Subsampling*	$\varvec{0.848\pm 0.082}$	$0.999\pm 0.051$
	*MT*	$0.843\pm 0.091$	$0.998\pm 0.066$
	*MT+*$\mathcal {L}_C$	$0.843\pm 0.084$	$\varvec{1.009\pm 0.065}$
$\times 3$	Bicubic	$0.654\pm 0.150$	$0.946\pm 0.167$
	Zero-filled	$0.782\pm 0.117$	$\varvec{1.009\pm 0.071}$
	UniRes	$\varvec{0.809\pm 0.198}$	$0.855\pm 0.271$
	*Subsampling*	$\varvec{0.809\pm 0.098}$	$0.967\pm 0.062$
	*MT*	$0.801\pm 0.104$	$0.999\pm 0.078$
	*MT+*$\mathcal {L}_C$	$\varvec{0.805\pm 0.100}$	$\varvec{1.010\pm 0.071}$
$\times 4$	Bicubic	$0.634\pm 0.158$	$0.835\pm 0.181$
	Zero-filled	$0.720\pm 0.142$	$0.955\pm 0.101$
	UniRes	$0.743\pm 0.210$	$0.851\pm 0.263$
	*Subsampling*	$\varvec{0.758\pm 0.117}$	$0.983\pm 0.069$
	*MT*	$\varvec{0.756\pm 0.120}$	$0.952\pm 0.104$
	*MT+*$\mathcal {L}_C$	$0.754\pm 0.118$	$\varvec{0.996\pm 0.088}$

**Table 5:** The results and their standard errors for motion artefacts. The three experiments differ in the percentage of the center of the *k*-space left uncorrupted for each corrupted image. The performance of the selected models is compared using regards to *SSIM* and *VIF*. The results in bold indicate the best performance without significant differences between them following a Nemenyi post-hoc test

		SSIM	VIF
$25\%$	Original	$\varvec{0.967\pm 0.036}$	$1.043\pm 0.058$
	TV	$\varvec{0.965\pm 0.036}$	$1.044\pm 0.056$
	*Motion*	$\varvec{0.963\pm 0.033}$	$1.017\pm 0.045$
	*MT*	$\varvec{0.959\pm 0.045}$	$1.031\pm 0.055$
	*MT+*$\mathcal {L}_C$	$\varvec{0.963\pm 0.034}$	$\varvec{1.046\pm 0.046}$
$12.5\%$	Original	$0.944\pm 0.064$	$1.015\pm 0.094$
	TV	$0.938\pm 0.064$	$\varvec{1.065\pm 0.091}$
	*Motion*	$\varvec{0.947\pm 0.045}$	$1.020\pm 0.046$
	*MT*	$0.937\pm 0.065$	$1.042\pm 0.069$
	*MT+*$\mathcal {L}_C$	$\varvec{0.945\pm 0.049}$	$1.047\pm 0.050$
$5\%$	Original	$0.908\pm 0.085$	$1.015\pm 0.111$
	TV	$0.903\pm 0.086$	$\varvec{1.1150\pm 0.108}$
	*Motion*	$\varvec{0.923\pm 0.059}$	$1.020\pm 0.048$
	*MT*	$0.910\pm 0.080$	$1.043\pm 0.080$
	*MT+*$\mathcal {L}_C$	$\varvec{0.921\pm 0.063}$	$1.046\pm 0.053$

**Table 6:** The results and their standard errors for denoising. The three experiments differ in the selected SNR ratio of the corruptions. The performance of the selected models is compared using regards to *SSIM* and *VIF*. The results in bold indicate the best performance without significant differences between them following a Nemenyi post-hoc test

		SSIM	VIF
5 dB	Original	$0.921\pm 0.070$	$1.023\pm 0.056$
	C.A.D.	$\varvec{0.961\pm 0.030}$	$\varvec{1.043\pm 0.032}$
	BM3D	$0.949\pm 0.033$	$1.017\pm 0.027$
	*Noise*	$\varvec{0.965\pm 0.029}$	$1.025\pm 0.032$
	*MT*	$0.957\pm 0.035$	$1.040\pm 0.051$
	*MT+*$\mathcal {L}_C$	$\varvec{0.958\pm 0.033}$	$\varvec{1.049\pm 0.048}$
0 dB	Original	$0.864\pm 0.107$	$1.006\pm 0.090$
	C.A.D.	$0.931\pm 0.049$	$\varvec{1.066\pm 0.044}$
	BM3D	$\varvec{0.941\pm 0.040}$	$1.035\pm 0.044$
	*Noise*	$\varvec{0.943\pm 0.041}$	$1.037\pm 0.038$
	*MT*	$\varvec{0.936\pm 0.049}$	$1.054\pm 0.059$
	*MT+*$\mathcal {L}_C$	$\varvec{0.935\pm 0.051}$	$1.057\pm 0.049$
$-5$ dB	Original	$0.771\pm 0.158$	$1.021\pm 0.113$
	C.A.D.	$0.874\pm 0.096$	$\varvec{1.099\pm 0.072}$
	BM3D	$0.850\pm 0.132$	$\varvec{1.092\pm 0.088}$
	*Noise*	$\varvec{0.906\pm 0.070}$	$1.066\pm 0.054$
	*MT*	$0.892\pm 0.081$	$1.063\pm 0.061$
	*MT+*$\mathcal {L}_C$	$0.886\pm 0.084$	$\varvec{1.091\pm 0.065}$

This is followed by the evaluations of scenarios where multiple artefacts are present, with the results in Table 7. For each image correction, the mean absolute values of the four residual outputs were collected for the multi-task models. For the first scenario, the values were [0.008, 0.032, 0.021, 0.004] and [0.005, 0.026, 0.010, 0.002] for the *MT* and *MT+*$\mathcal {L}_C$ models respectively. For the second scenario, the values were [0.026, 0.002, 0.012, 0.058] and [0.010, 0.002, 0.006, 0.060] for the two models.

**Table 7:** The results and their standard errors for multi-task. The first block shows the results for corrupting the original images by subsampling the *k*-space and applying motion artefacts. The second block shows the results for correcting images that are corrupted by noise and bias fields as well. The results in bold indicate the best performance without significant differences between them following a Nemenyi post-hoc test

	SSIM	VIF
Original	$0.625\pm 0.162$	$0.905\pm 0.075$
*Subsampling*	$0.662\pm 0.138$	$0.953\pm 0.076$
*Subsampling* + *Motion*	$0.633\pm 0.157$	$0.921\pm 0.082$
*Motion*	$0.678\pm 0.129$	$0.919\pm 0.076$
*Motion* + *Subsampling*	$0.631\pm 0.159$	$\varvec{0.963\pm 0.074}$
*MT*	$0.672\pm 0.148$	$0.930\pm 0.107$
*MT+*$\mathcal {L}_C$	$\varvec{0.692\pm 0.137}$	$0.941\pm 0.080$
Original	$0.380\pm 0.080$	$0.845\pm 0.070$
*Bias*	$0.399\pm 0.072$	$0.810\pm 0.075$
*Bias* + *Noise*	$0.764\pm 0.137$	$\varvec{1.010\pm 0.049}$
*Noise*	$0.788\pm 0.113$	$1.009\pm 0.049$
*Noise* + *Bias*	$0.420\pm 0.072$	$0.832\pm 0.065$
*MT*	$0.792\pm 0.106$	$0.986\pm 0.048$
*MT+*$\mathcal {L}_C$	$\varvec{0.796\pm 0.115}$	$\varvec{1.007\pm 0.049}$

The performance of the *Pelvis MT+*$\mathcal {L}_C$ model, using two examples from the Gold Atlas dataset is visualized on Fig. 2.

## Discussion

The Bayesian hyper-parameter optimization shows that all models improve by using regularization, with the best values ranging between 0.48 and 0.89. Both models using $\mathcal {L}_C$ favor a higher $\alpha$, 0.87 and 0.89 for the brain and pelvic datasets, respectively.

Both models used a small scaling factor for the identity kernel, $I_E$, namely 0.30 and 0.13, respectively, which means the *MSE* loss contributed only a small fraction of the final loss. The models favored the $3\times 3$ top Sobel operator over the $5\times 5$ version, while favoring the $5\times 5$ version of the right Sobel operator over the $3\times 3$ kernel. Similarly, the $5\times 5$ Laplace kernel had a much larger contribution to the loss than the $3\times 3$ kernel. The largest difference between the $\delta$ values of the two models was the top Prewitt operator, which had a much larger scaling factor for the *MT+*$\mathcal {L}_C$ model. This might be due to the fact that this model has a smaller $\delta$ for the other top edge detection kernels, than the *Pelvis MT+*$\mathcal {L}_C$ model. In general, out of the kernels performing similar tasks, only one of them contributed largely to the loss, while all other $\delta$ values were minimized.

Despite using different training datasets of different anatomies, the two models implementing $\mathcal {L}_C$, *MT+*$\mathcal {L}_C$ and *Pelvis MT+*$\mathcal {L}_C$ found similar $\delta$ values to work best for the individual kernels. From these findings, we propose that the Bayesian optimization approach does not have to be repeated to find the optimal $\mathcal {L}_C$, instead the $\delta$ values can be adjusted to the values presented here.

For bias field correction, the less pronounced fields ($5\%$) have a larger effect on *SSIM*, than on the *VIF* metric. While the *Bias* and *MT* models achieve the best *SSIM* results, but not for *VIF*, and the ‘N4ITK’ method achieves the best *VIF* results, but not for *SSIM*, the *MT+*$\mathcal {L}_C$ model performs well regarding both evaluation metrics.

For *k*-space subsampling, the trained models outperformed bicubic interpolation and the UniRes model, with regards to *VIF*. The proposed models can handle a wider variation of subsampling masks than the ones evaluated here.

**Fig. 1:**
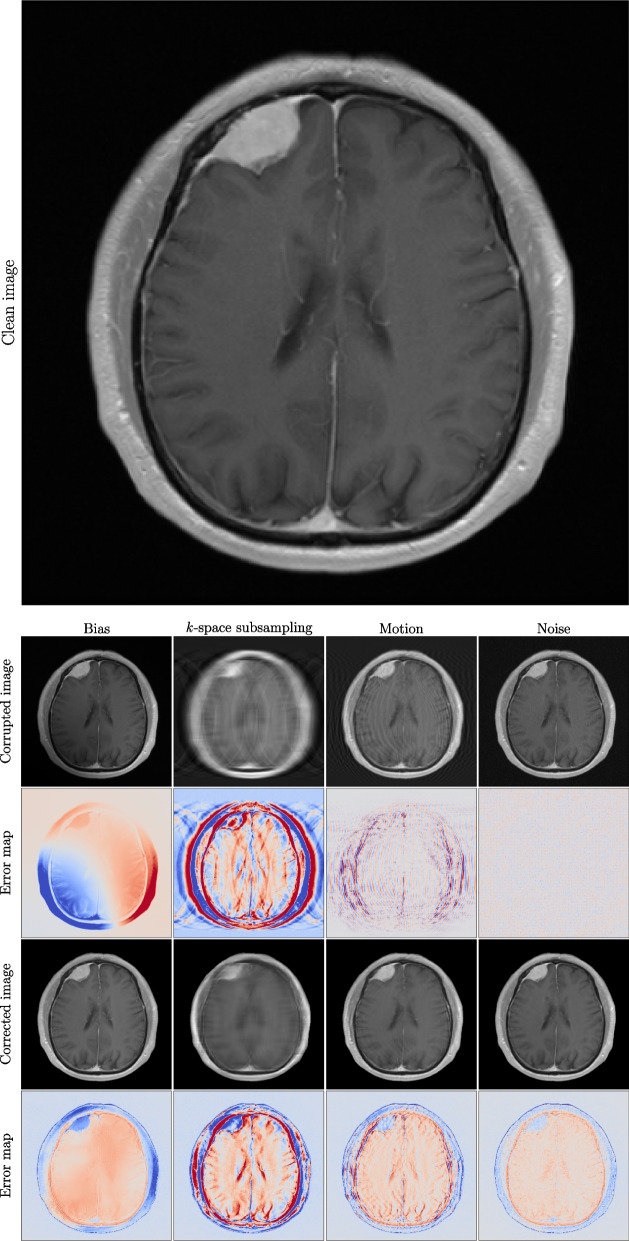
Artefact augmentations and their corrections by the *MT+*$\mathcal {L}_C$ model for an example image (seen in the top row). The second row shows the artefacted images, while the fourth row shows their correction by the trained model. The figure shows examples for all artefact types from left to right: bias, *k*-space subsampling, motion, and noise. For each artefact type, the images and their difference to the original clean image are shown in the third and fifth row, respectively

For motion correction, the smaller motions introduced only a small change with regards to *SSIM* which could not be significantly improved by either of the methods. For larger motions, although the trained models achieved the best results with regards to *SSIM*, the Total Variation denoising achieved the best *VIF* results. Looking at an example correction of the model in Fig. 1, we can see that the model removes a large amount of ripple effects and retains the sharp edges of the clean image.

While the models trained for correcting noise generally perform best with regards to *SSIM*, only the *MT+*$\mathcal {L}_C$ model shows comparable results to C.A.D. and BM3D with regards to *VIF*.

For an example image slice, all augmented artefacts are visualized, and their respective corrections by the *MT+*$\mathcal {L}_C$ model in Fig. 1. While the error maps of the motion and noise corrections display higher values compared to the original images, both examples still demonstrate an improvement in the *SSIM* and *VIF* results. In the case of the error maps for additive noise corrections, the blue contour surrounding the skull indicates a decrease in the pixel values relative to the original image. However, this difference was not perceptible upon visual inspection.

Evaluating the performance of $\mathcal {L}_C$, we see that for all individual artefact correction tasks, the *MT+*$\mathcal {L}_C$ model has always reached a higher *VIF* score than the *MT* model.

Regarding the multi-task aspect of the model, contrary to our hypothesis, the performance of the models trained to correct only a single artefact type did not decrease in the presence of another, previously unseen artefact. In fact, for both cases where two different artefacts were applied on an image (*k*-space subsampling and motion, bias and noise) a combination of two alternative models could achieved similar, or even better performance than our multi-task models. However, the order of applying the models changes the performance significantly. For the case of *k*-space subsampling and motion, applying the *Motion* model first, and the *Subsampling* model second achieved the best *VIF* results, however applying the models in the other order achieves significantly worse results than our multi task models. For the other scenario, applying the *Bias* model first, and then the *Noise* model yields significantly better results than the other order. A possible reason for the superior results of combining two models compared to the multi-task model stems from the complexity of the two methods. Since all models use the same network architecture, the combination of the two models has double the number of model parameters, compared to the proposed multi-task solution. However, the large difference in performance when changing the order of the two models in the consecutive approach, also suggests that increasing the complexity of the approach not only leads to an increase in performance but also in sensitivity.

The mean absolute values of the residuals show that the largest corrections were indeed added to the artefacts that were present in the images.

In the second scenario, the *Noise* model seems especially sensitive to bias, which could be explained by the model assuming a homogeneous pixel intensity within the tissues to estimate the noise.

The *MT+*$\mathcal {L}_C$ model significantly outperformed *MT* in both scenarios, with regards to both evaluation metrics.

**Fig. 2:**
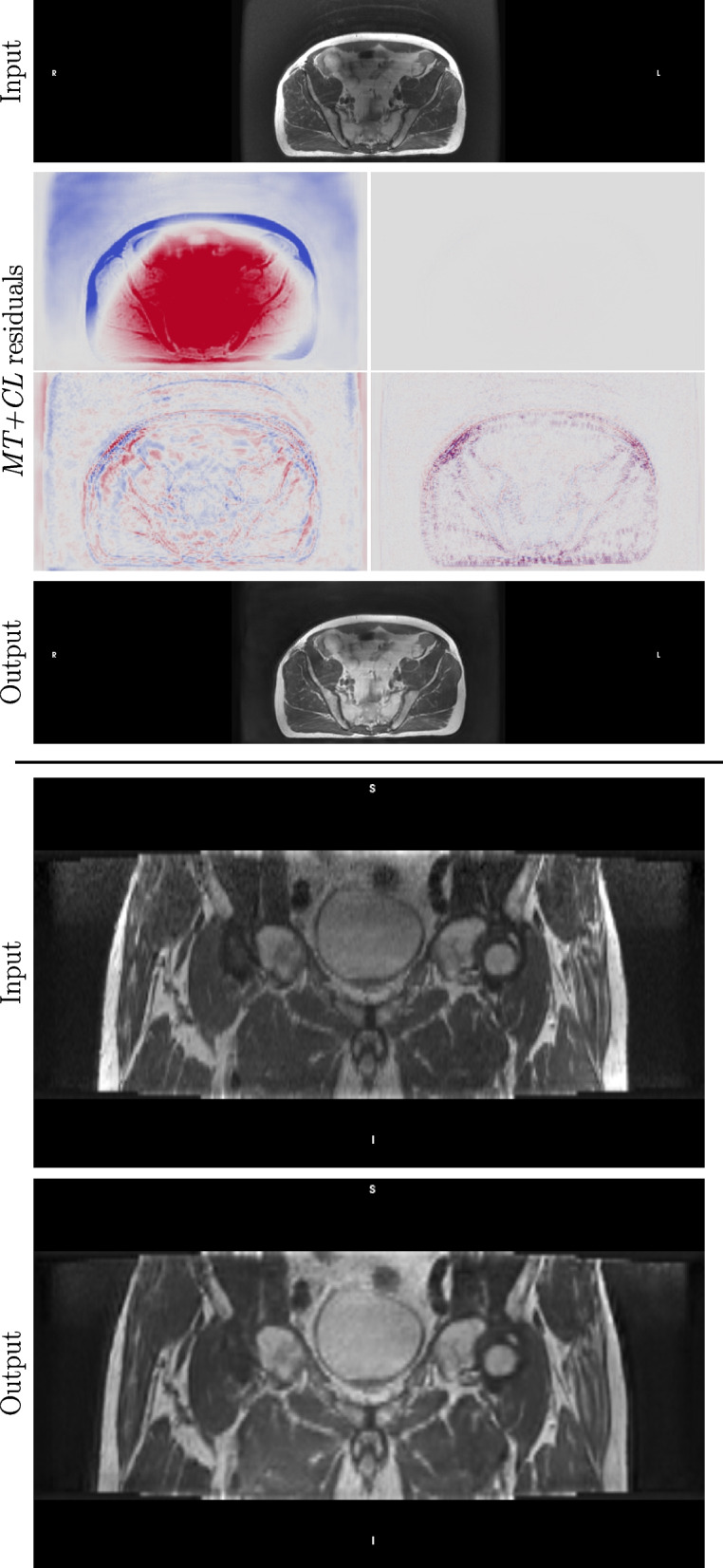
Example corrections using the model *Pelvis MT+*$\mathcal {L}_C$ for pelvic MRI scans from Gold Atlas of two patients. The top part shows a slice from the first patient with the input slice (top), the prediction (bottom) and the output residuals for bias, subsampling, motion and noise, respectively. The bottom part shows another scan of 77 axial slices from the coronal view (top) and the corresponding corrections by the model (bottom)

The example corrections of pelvic scans in Fig. 2 show that the residuals indicate well the artefacts they correspond to. The bias term is generally smooth and slowly varying, while the subsampling term is insignificant since the input image was already of full-resolution (all residuals are plotted using the same scale). The motion term shows ripple effects that are corrected for in the reconstructed output.

The second example illustrates how the model removes bias and noise from the image slices to return a cleaner image, and it also shows how the axial, slice-wise corrections do not introduce artefacts when viewed from an orthogonal angle.

## Conclusions

We have developed and trained two multi-task, deep learning models using our novel loss function, for artefact correction in brain, and pelvic MR data. It provides more robust results than using a combination of machine learning solutions correcting only a single artefact, and it also performs all four tasks with similar or superior performance to other, established methods.

Our work included implementing the augmentation of four of the most typical artefacts in MR data, bias fields, subsampled *k*-space, motion, and noise. The model simultaneously corrects all four aforementioned artefact types, without any decrease in performance compared to models trained for correcting only one artefact type.

The multi-task approach also proved more robust in the scenario of multiple simultaneous artefacts, compared to applying the individual models sequentially.

Our proposed convolutional loss function introduces the differences in the image gradients in the computed loss, through the use of image convolutions. The introduction of the Laplace operator, and at least one top and right edge detection kernels improve the performance of the model for artefact corrections in all cases with regards to *VIF*. The thorough evaluation of the model trained on brain scans showed to outperform several alternative methods for the correction of individual artefacts.

In addition, our example corrections on pelvic scans indicated that the performance of the model translates well to real data despite being trained on augmented artefacts.

To improve the reproducibility of our approach, we have made all trained models and source code openly accessible.

## Data Availability

The fastMRI dataset [29, 30] (https://fastmri.med.nyu.edu/) is publically available. For evaluating the pelvic model, we have used the publicly available Gold Atlas dataset [31] (https://zenodo.org/record/583096). The source code used for the conclusions of this article is available on Github (https://github.com/attilasimko/mri-ac). The trained models are also available in Hero Imaging (https://heroimaging.com), in its “AI Model Zoo” collection. We used TensorFlow with two RTX 3090 and four 2080 Ti GPUs for training.

## Footnotes

1 https://fastmri.med.nyu.edu/

2 https://zenodo.org/record/583096

3 https://github.com/brudfors/UniRes

4 http://www.simpleitk.org

5 https://pypi.org/project/bm3d/
